# LRRK2 G2019S reprograms innate and adaptive immunity to drive context-dependent host defense outcomes

**DOI:** 10.3389/fncel.2026.1860967

**Published:** 2026-07-29

**Authors:** Andrea R. Merchak, Mary K. Herrick, Madelyn C. Houser, Dina A. Moustafa, Joanna B. Goldberg, Jeremy M. Boss, Jacob Kohlmeier, Darcie M. Cook, Cody E. Keating, Jianjun Chang, Andrew B. West, Malú Gámez Tansey

**Affiliations:** 1 Department of Neuroscience, University of Florida College of Medicine, Gainesville, FL, United States; 2 Department of Neurology, Stark Neuroscience Research Institute, Indiana University School of Medicine, Indianapolis, IN, United States; 3 Department of Physiology, School of Medicine, Emory University, Atlanta, GA, United States; 4 Center for Data Science, Nell Hodgson Woodruff School of Nursing, Emory University, Atlanta, GA, United States; 5 Division of Pulmonary, Asthma, Cystic Fibrosis, and Sleep, Department of Pediatrics, Emory University School of Medicine, Atlanta, GA, United States; 6 Department of Microbiology and Immunology, Emory University School of Medicine, Atlanta, GA, United States; 7 Duke Center for Neurodegeneration and Neurotherapeutic Research, Department of Pharmacology and Cancer Biology, Durham, NC, United States

**Keywords:** infection, inflammation, LRRK2 G2019S, myeloid cells, T cells, monocytes

## Abstract

**Introduction:**

Parkinson’s disease (PD) is increasingly recognized as a neurological disorder characterized not only by neurodegeneration but also by chronic immune dysregulation across the lifespan. Although the initiating events underlying PD remain unclear, accumulating evidence suggests that inflammatory processes may contribute to disease susceptibility and progression. Mutations in leucine-rich repeat kinase 2 (*LRRK2*), particularly the gain-of-function G2019S variant, represent the common genetic cause of familial PD and have been implicated in immune regulation and infection susceptibility. To date, most research has focused on the effect of *LRRK2* mutation in neurons and the contributions of G2019S-mediated kinase activity to neuronal toxicity, leaving the role of G2019S-mediated kinase activity in immune cell homeostasis and its contribution to PD pathogenesis largely unresolved.

**Methods:**

Here, we used murine overexpression models of wildtype and G2019S variant of mouse *Lrrk2* to examine how *Lrrk2* G2019S shapes host defense across viral and bacterial infection models. We tested systemic sepsis and *Escherichia coli* infection to examine bacterial clearance. We followed up by testing macrophage responses to intracellular (Listeria monocytogenes) or primarily extracellular (Pseudomonas aeruginosa) bacteria. Finally, tested antibody-mediated immunity using influenza and cytotoxic T cell-mediated immunity using lymphocytic choriomeningitis virus (LCMV) infection.

**Results:**

We found that *Lrrk2* G2019S overexpression enhanced survival and bacterial clearance during P. aeruginosa lung infection, whereas the same genotype worsened outcomes in polymicrobial sepsis, with increased mortality, pulmonary myeloid infiltration and a hematopoietic cell-intrinsic phenotype. During L. monocytogenes infection, *Lrrk2* G2019S selectively reduced non-classical monocytes without altering disease progression. In influenza and LCMV infection, G2019S altered antigen-specific CD8^+^ T cell distribution without major changes in cell memory responses clinical severity.

**Discussion:**

Together, these data show that *Lrrk2* G2019S selectively reprograms innate and adaptive immunity in a pathogen- and tissue-dependent manner, uncoupling inflammatory magnitude from effective host defense. These results and previous work support a model in which the G2019S *LRRK2* variant contributes to maladaptive inflammatory responses to specific infection challenges, providing insight into how lifetime immune perturbations may intersect with genetic susceptibility to influence lifetime infection risk.

## 1 Introduction

Leucine-Rich Repeat Kinase 2 (LRRK2) is one of the strongest genetic contributors to Parkinson’s disease (PD). The pathogenic G2019S mutation of *LRRK2* increases kinase activity and has been linked to changes in vesicular trafficking, cytoskeletal regulation and inflammatory signaling (West et al., 2005, 2007; Greggio et al., 2006). LRRK2 regulates multiple cellular processes including neurite outgrowth, cytoskeletal maintenance, and autophagy, all of which are disrupted by mutations in *LRRK2* (Plowey et al., 2008; Ramonet et al., 2011; Berwick and Harvey, 2012; Schapansky et al., 2014). To date, most research has focused on the effect of *LRRK2* mutation in neurons and the contributions of G2019S-mediated kinase activity to neuronal toxicity, leaving the role of G2019S-mediated kinase activity in immune cell homeostasis largely undefined.

LRRK2 expression is higher in immune cells than neurons, suggesting LRRK2 has an important role in the immune system (West, 2017). LRRK2 protein is found in adaptive (B and T cells) and innate (dendritic cells, macrophages, and monocytes) immune cells, and its expression is increased upon stimulation. However, the exact function of LRRK2 in these cells is unknown (Gardet et al., 2010; Hakimi et al., 2011; Thévenet et al., 2011; Atashrazm et al., 2019; Merchak et al., 2026). Intriguingly, higher levels of LRRK2 protein are present in adaptive and innate immune cells of idiopathic PD patients compared to healthy controls, underscoring the importance of investigating the contribution of LRRK2 to immune cell function (Cook et al., 2017).

Gene expression from tuberculosis infections identified LRRK2 as being significantly enriched during active infections (Wang et al., 2018). *Lrrk2* knockout macrophages treated with *Mycobacterium tuberculosis* exhibited increased IL-6, TNF, and IFNγ and proinflammatory gene transcription induced by mitochondrial stresses (Härtlova et al., 2018; Weindel et al., 2020, 2022). Phagosome maturation and bacterial control were increased in human and mouse macrophages treated with LRRK2 kinase inhibitors, suggesting that reduced LRRK2 kinase activity is protective against *M. tuberculosis* (Härtlova et al., 2018). On the other hand, *Lrrk2* knockout mice infected with *Salmonella typhimurium* exhibited reduced caspase-1 activation and IL-1β secretion due to inflammasome activation in macrophages, which ultimately impaired clearance of pathogens (Liu et al., 2017; Zhu et al., 2025). A recent study suggested that wildtype LRRK2 is protective against *S. typhimurium* and reovirus infection in a sex-dependent manner with females exhibiting impaired ability to control infection (Shutinoski et al., 2019). Interestingly, mice with the G2019S variant of *Lrrk2* controlled *S. typhimurium* infection better than wildtype mice but were more susceptible to reovirus in a LRRK2 kinase-dependent manner (Shutinoski et al., 2019). Further, the G2019S variant also increased immunopathology in response to *Citrobacter rodentium* infection, modulating neutrophil influx and Th17 responses (Ahmadi Rastegar et al., 2022). Collectively, these studies suggest a role for LRRK2 in immunity to control infection burden; however, the genotype- and infection-specific responses remains to be elucidated, underscoring the complexity of LRRK2 signaling and function. We therefore asked whether *LRRK2* G2019S alters innate and adaptive immunity in distinct infection settings and whether these effects are kinase dependent.

## 2 Materials and methods

### 2.1 Animals

All experimental procedures involving use of animals were performed in accordance with the National Institutes of Health Guidelines for Animal Care and Use. The studies were carried out in strict accordance with established guidelines and policies at Emory University School of Medicine or the University of Alabama, and recommendations in the Guide for Care and Use of Laboratory Animals, as well as local, state, and federal laws.

Hemizygous male and female bacterial artificial chromosome (BAC) transgenic mouse strains overexpressing either mouse mutant G2019S *Lrrk2* (G2019S) or mouse wildtype *Lrrk2* (WTOE) as well as non-transgenic littermate controls (B6) were used in all experiments. Homozygous male BAC *Lrrk2-G2019S* (B6.Cg-Tg(Lrrk2*G2019S)2Yue/J; stock number 012467) and BAC *Lrrk2-WT* (B6.Cg-Tg(Lrrk2)6Yue/J; stock number 012466) transgenic mice were purchased from the Jackson Laboratory and bred to hemizygosity at Emory University (protocol # DAR-2003358-122818BN) or the University of Alabama at Birmingham (protocol# PROTO201700441). Hemizygous male and female BAC transgenic mouse strains overexpressing either mouse mutant G2019S *Lrrk2* (G2019S) or mouse wildtype *Lrrk2* (WTOE) were used for experimental procedures with non-transgenic littermates on the same genetic background (B6/J) serving as controls. LRRK2 knockout (*Lrrk2^–/–^*) (C57BL/6-*Lrrk2^*TM*1.1*Mjff*^*/J; stock number 016121) mice were used in the CLP model. Bone marrow transplantations were performed using congenic (expressing CD45.1/Ly5.1 marker) mice as recipients (B6.SJL-Ptprca Pepcb/BoyJ obtained from Jackson Laboratories). Genotypes were determined by tail-snip PCR with two sets of primers: Transgene: Forward 5′ GAC TAC AAA GAC GAT GAC GAC AAG 3′ Reverse 5′ CTA CCA CCA CCC AGA TAA TGT C 3′; Internal positive control: Forward 5′ CAA ATG TTG CTT GTC TGG TG 3′ Reverse 5′ GTC AGT CGA GTG CAC AGT TT 3′. Quantitative PCR was used to determine the copy number of the BAC transgene from these transgenic mice. The mice used in these experiments had 20–30 copies of the BAC transgene. Animals were group-housed (maximum 5 mice per cage) and maintained on a 12 h/12 h light/dark cycle with *ad libitum* access to standard rodent chow and water.

### 2.2 Murine infection models

Cecal Ligation and Puncture (CLP) Sepsis Model: CLP surgeries were performed in blinded fashion aseptically under isoflurane anesthesia as described previously (Toscano et al., 2011). Briefly, exposed cecum through midline abdominal incision was ligated 40%–50% of its total length (measured from the ileo-cecal valve to the distal tip/end of the cecum) using 4-0 silk suture. 21-gauge needle was used to puncture (2 holes) the ligated cecum. A small amount of fecal matter was squeezed gently through the holes in the cecum. Cecum was placed back in the peritoneal cavity and the closure was obtained with 5-0 Prolene sutures. Cecum was exposed out and put back in the peritoneal cavity for the no CLP/sham control mice. Mice were administered 1 mL 0.9% normal saline and buprenorphine (0.05 mg/kg of body weight; analgesic) post-surgeries. Mice were observed for survival (5 days) or were euthanized 18 h post CLP for subsequent studies.

*Pseudomonas aeruginosa:* Murine pneumonia model studies were performed according to the method previously described (Moustafa et al., 2023). *P. aeruginosa* strain PAO1 P1-*lux* (Damron et al., 2013) was grown on *Pseudomonas* isolation agar (PIA; BD Biosciences) for 16–18 h at 37 °C and suspended in PBS to an optical density at 600 nm (OD_600_) of 0.5, corresponding to ∼109 CFU/ml. Bacterial inoculum was adjusted spectrophotometrically to obtain the desired sublethal (≤0.5 LD_50_) challenge dose in a volume of 20 μl. Animals were anesthetized by intraperitoneal injection of 0.2 ml of a mixture of ketamine (6.7 mg/ml) and xylazine (1.3 mg/ml) and then infected via intranasal (IN) route with total volume of 20 μl (10 μl/nostril; young mice 3–4-month-old: 1.75 × 10^6^ CFU/mouse, aged mice 20–22 month-old: 4 × 10^6^ CFU/mouse). Mice were either euthanized at indicated time point(s), or imaged and monitored for survival.

Influenza: Intranasal inoculation with influenza A/PR/8/34 (H1N1; ATCC; VR-95) at 3000 plaque-forming units (PFU) was performed with mice under isoflurane anesthesia.

LCMV: Lymphocytic Choriomeningitis Virus (Armstong strain; ATCC; VR-127). Mice were infected by intraperitoneal injection with 2 × 10^5^ PFU.

*Listeria monocytogenes (LCMV)*: Mice were inoculated with by intravenous injection with 2 × 10^3^ PFU *L. monocytogenes* (strain 45231).

Mice were monitored with daily weighing and euthanized if they reached 25% weight loss.

### 2.3 PF-360 administration

PF-360 was prepared in 0.5% methylcellulose (Sigma) solution. Mice treated with 20 mg/kg of the selective LRRK2 kinase inhibitor PF-360 (generously provided by Pfizer) or vehicle were dosed by oral gavage twice daily for 14 days post infection. Short term cohorts were euthanized at 8- or 10- days post infection while LCMV and influenza long term analysis was conducted after euthanasia at 30- or 35-days post infection, respectively. Oral gavages were done in blinded fashion.

### 2.4 Bone marrow transfer

Bone marrow transplantations (BMT) were performed using congenic (expressing CD45.1/Ly5.1 marker) mice as recipients (B6.SJL-Ptprca Pepcb/BoyJ obtained from Jackson Laboratories) (Parks et al., 2009). Myeloablation of these recipient mice (3 months old) was achieved by using ionizing radiation (two equal split doses spaced by 3 h; a total of 9–11 Gy). All recipient mice were transplanted with 3–5 × 10^6^ bone marrow mononuclear cells from either G2019S mice (expressing CD45.2/Ly5.2 marker) or nTg (expressing CD45.2/Ly5.2 marker) donor mice. Transplanted mice were maintained on antibiotics/bleach water for 4 weeks. Reconstitution of blood leukocytes post BMT was evaluated by flow cytometry (antibodies information in Flow cytometry section) on blood obtained from retro orbital puncture. Blood leukocyte repopulation was >90% in all transplanted mice. After BMT, 94.3 ± 0.7% and 93.5 ± 0.8% of the blood leukocytes in mice receiving nTg and G2019S bone marrow, respectively, were CD45.2/Ly5.2 positive; % reconstitution expressed as % of total live CD45^+^ cells.

### 2.5 Tissue collection

Mice were euthanized with avertin (2,2,2-Tribromoethanol, Sigma) and exsanguinated prior to tissue harvest. Tissues were collected and single-cell immune cell populations isolated as previously described (McMaster et al., 2015; Dunbar et al., 2020; Hayward et al., 2020).

Lung from *P. aeruginosa* infected mice: In sum, bronchioalveolar lavage was performed, and the airways were washed five times with HBSS (Gibco; 14175079) after which the lungs were harvested into HBSS. Lungs were finely minced using a razor blade and digested in collagenase D (Roche; 1108885800) for 30 min at 37 °C. Cells were centrifuged for 5 min at 400 *g* at room temperature. Pellet was resuspended in 4 mL 40% Percoll (Millipore Sigma; P1644) HBSS solution. Beneath this, 4 mL of an 80% Percoll HBSS solution was underlaid. The Percoll gradient was centrifuged for 30 min at 400 *g* at room temperature without brake. The cell layer was isolated, washed, and resuspended in PBS.

Lung from CLP mice: Lungs harvested from ice cold, saline-perfused mice were finely minced using a razor blade and digested in Liberase DL enzyme (1.67 U/mL; Roche Diagnostics) in Dulbecco’s modified Eagle’s medium for 30 min at 37 °C. The digested homogenate was passed through a series of syringe needles (15, 18, and 21-guage) and through a 40 μm nylon filter (Fisher Scientific) to obtain single cell suspension. Cells were centrifuged for 10 min at 1670 *g* at 4 °C. Pellet was lysed for 5 min at room temperature using 1x red blood cell lysis buffer (BioLegend; 420301) and centrifuged for 10 min at 1670 *g* at 4 °C. The cells were washed and resuspended in PBS.

Blood: Whole blood was collected by submandibular bleeds or cardiac puncture with approximately 200 μl per mouse collected for each time point. Whole blood was collected in ethylenediaminetetraacetic acid (EDTA)-coated tubes (Covidien 8881311248). 50 μL of whole blood was treated with 1x red blood cell lysis buffer (BioLegend; 420301) to lyse red blood cells per the manufacturer’s instructions prior to staining PBMCs for flow cytometry. The remaining 150 μL of whole blood was centrifuged after which plasma was removed and promptly frozen on dry ice then subsequently stored at −80 °C until processing.

Spleen and Mediastinal Lymph Node: Splenocytes and lymph nodes were obtained by mechanical dissociation and straining through nylon mesh. Red blood cells in splenocytes were lysed with buffered ammonium chloride.

### 2.6 Bioluminescent imaging

For animals infected with either *P. aeruginosa* PAO1 P1-lux, or injected with Xen14 bioluminescent *E. coli* intraperitoneally (i.p.). Colonization and localization of bioluminescent bacteria was monitored in real time using an IVIS Lumina LT III imaging system (PerkinElmer) according to the method previously described (Moustafa et al., 2021). Briefly, each group of mice was anesthetized with 3% isoflurane using an XGI-8 gas anesthesia system (Caliper Life Sciences), and imaged using medium binning, f/stop 1, subject height 1.5 cm. Images were acquired with up to 5-min exposures. Total photon emission from the ventral and dorsal sides of imaged mice were quantified using Living Image Software v4.0x (Xenogen Corp.). All correlations were done as average radiance of photons emitted per second, area, and steradian (p/s/cm^2^/sr) under the chosen experimental conditions. Bioluminescent imaging was conducted at 24- and 48-h post infection.

### 2.7 Lung bacterial counts

Aged mice infected with *P. aeruginosa* were euthanized 24 h after infection. Whole lungs were collected aseptically, weighed, and mechanically homogenized in 1 mL of phosphate buffered saline. Tissue homogenates were serially diluted and plated on PIA. CFU determinations were made after 18 h of incubation at 37 °C.

### 2.8 Flow cytometry

Cecal ligation and puncture and *E. coli* models: The leukocytes obtained (lung/blood) were stained for 30 min on ice with the following anti-mouse antibodies conjugated to fluorophores (with their cat #) appropriate for flow cytometry: 7-aminoactinomycin D (00-6993-50) for live-dead staining; phycoerythrin-conjugated (PE) CD64 (558455), NK 1.1 (12-5941-82), CD11b (12-0112-82); allophycocyanin-conjugated (APC) Gr-1 (17-9668-82), CD3e (17-0031-82), CD19 (17-0193-82), CD45.1 (EB/17-0453-82); Fluorescein isothiocyanate-conjugated (FITC) CD45 (11-0454-82), MHCII (115321-85), CD45.2 (EB/11-0454-82); CD8 eF450(48-0081-82); Ly6C eF450(48-5932-82); CD4 eVolve605 (83-0042-42); CD11b eVolve605 (83-0112-42) CD45.2 BV650 (109836); CD11c BV785 (117336); F4/80 APC eF780 (47-4801-82); CD19 APC eF780 (47-0193-82). All these reagents were obtained from eBioscience, Biolegend, BD Pharm, Fisher Scientific. For obtaining absolute numbers Accucheck counting beads (PCB-100, Life technologies) were used as per manufacturer’s instructions. After staining and washing, cells were analyzed on a LSRII (BD Bioscience, San Jose, CA). Isotype-matched, fluorescently conjugated antibodies of irrelevant specificity were used as controls. Results were analyzed using FlowJo Software (Tree Star Inc., Ashland, OR). The gating strategy for myeloid and lymphoid populations for blood and lung are shown in Supplementary Figure 1.

*Listeria monocytogenes*, Influenza, and LCMV models: Single-cell populations were isolated in PBS as described above. Cells were stained with Live/Dead Zombie Yellow, Ultraviolet, or Near Infrared (Biolegend) and incubated with anti-mouse CD16/CD32 (eBioscience; 14-0161-85). Antibodies include anti-mouse CD11b BV711 (Biolegend), anti-mouse LyG BV510 (Biolegend), anti-mouse Ly6C APC-Cy7 (BD Biosciences), anti-mouse CCR2 APC (R&D), anti-mouse B220 PE-Cy7 (Biolegend), anti-mouse CD3 APC (Biolegend), anti-mouse CD4 APC-Cy7 (eBiosciences), anti-mouse CD8 BV750 (Biolegend), anti-mouse CD44 AF700 (eBiosciences), anti-mouse KLRG-1 BV605 (Biolegend), anti-mouse CD127 PE-CF594 (BD Biosciences), anti-mouse CD62L BV605 (Biolegend), anti-mouse CD69 PE-Cy7 (Biolegend), and anti-mouse CXCR3 BV650 (Biolegend). Tetramers used for detection of antigen-specific cells included H-2Kb Listeria OVA (eBiosciences), H-2Db Influenza A NP366-374 (ASNENMETM), and H-2Db LCMV GP33 (KAVYNFATM) with the latter two supplied by the NIH tetramer core at Emory. Samples were run on an LSR Fortessa or LSRII (BD Biosciences) and analyzed with FlowJo_V10. Events were gated on singlets and live cells. Additional CD3^+^ gating was used for T cells. The terminal gating strategy is shown in the figures.

### 2.9 Multiplex immunoassay

Levels of inflammatory protein were measured in plasma by the Emory Multiplexed Immunoassay Core (EMIC) using a commercially available V-plex Pro-inflammatory Panel 1 Mouse Kit per the manufacturer’s instructions on the Meso Scale Discovery113 QuickPlex (Meso-Scale Discovery, Gaithersburg, MD). Plasma samples were analyzed for cytokines and chemokines (IFNγ, IL-1β, IL-2, IL-4, IL-5, IL-6, IL-10, IL-12p70, KC/GRO, and TNF) that play key roles in the inflammation response and immune system. Samples were run in replicates of 30 μL by an experimenter blinded to treatment history and genotype.

### 2.10 Western blot

Immunoblotting was performed as previously described (de Sousa Rodrigues et al., 2016). Bicinchoninic acid (BCA) protein assay (Pierce Scientific, 23225) was used to determine protein concentrations, after which lysates were further diluted 1:1 with 2x Laemmli buffer (BioRad, 1610737) and boiled at 90 °C for 5 min. Samples (10 μg) were electrophoresed using 4%–20% Mini-PROTEAN TGX precast gels (BioRad, 4561096) and transferred to 0.45 μm PVDF membranes using the Trans-Blot Turbo System (BioRad, 1704150EDU) according to the manufacturer’s protocol. Membranes were washed and then incubated in 5% powdered milk blocking buffer (BioRad, 1706404) for 1 h before applying primary antibody overnight at 4 °C. The following morning, membranes were briefly washed and incubated at room temperature with HRP-conjugated secondary antibodies for 1 h. Membranes were briefly washed and imaged using Azure Biosystems C400 system to detect chemiluminescent signal. Bands were quantified by densitometry using ImageStudio Lite software. Protein expression was normalized to total protein on a Li-Cor Odyssey instrument (Li-Cor #926-11015).

### 2.11 Statistical analysis

In all experiments, mice were randomly assigned to treatment groups. Survival, weighing, and downstream analysis were conducted in a blinded fashion and then decoded after the collection of the data. Immune cell populations and percent body weights were compared across genotype and treatment using two-way analysis of variance (ANOVA) or mixed-effects model with GraphPad Prism 8 software. Tukey’s multiple comparisons test was used for *post hoc* analysis. Survival curves were compared using Kaplan Meier Survival Analysis with Log-rank test to identify differences in groups. Colonization was compared across genotype using one-way ANOVA with Tukey’s *post hoc* analysis. Weight, multiplexed immunoassay, and PMBC flow data were compared across genotypes and time, while immunoblot was compared across genotype and treatment using two-way analysis of variance (ANOVA) or mixed-effects model with Tukey’s multiple comparisons test. Data points were excluded if they were statistical outliers or in the case of flow cytometry- if insufficient cells were collected due to technical error. Significance for all statistical comparisons was set at *p* < 0.05. All data are presented as mean ± SEM. Letters and asterisks above groups signify *post hoc* results. Groups sharing the same letter were not significantly different.

## 3 Results

To investigate the role of LRRK2 in immune regulation and determine whether LRRK2 levels or its gain-of-function kinase activity alter immune cell function, we compared normal B6 mice to bacterial artificial chromosome transgenic mice overexpressing mouse wildtype *Lrrk2* (WTOE) or mutated G2019S *Lrrk2* (G2019S). Mice were inoculated with viral (lymphocytic choriomeningitis virus (LCMV) and influenza virus) and bacterial [*Escherichia coli (E. coli), Listeria monocytogenes (L. monocytogenes)* and *Pseudomonas aeruginosa (P. aeruginosa)*] infections in the presence or absence LRRK2 kinase inhibition (PF-360). Utilization of these particular infections in the same LRRK2 mouse models permitted assessment of specific arms of the immune system, with a focus on antibody-mediated immunity (influenza), CD8 T cells (LCMV), and macrophage responses to intracellular (*L. monocytogenes*) or primarily extracellular (*P. aeruginosa*) bacteria.

### 3.1 G2019S enhances clearance of *P. aeruginosa* infection

**FIGURE 1:**
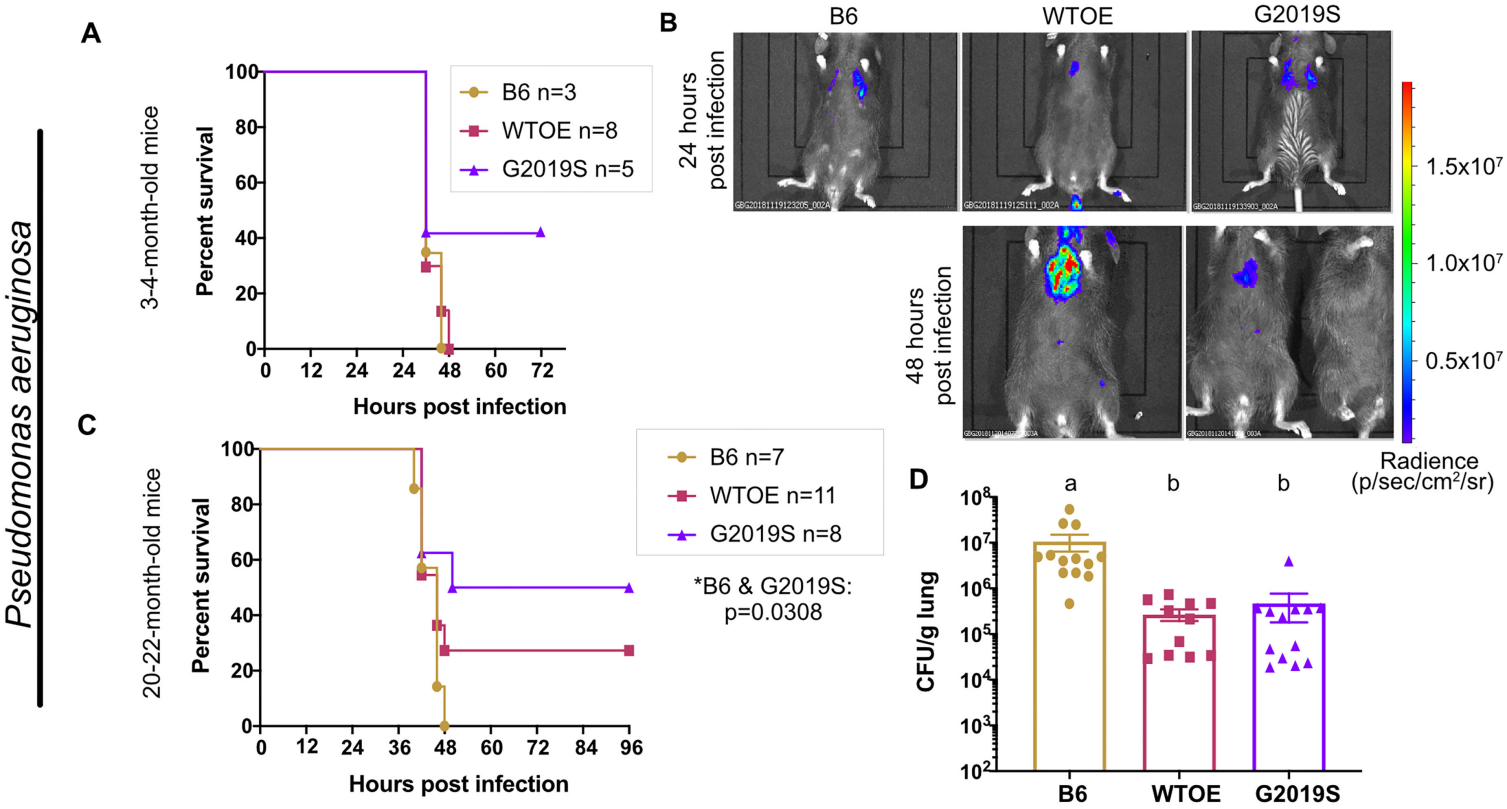
*Lrrk2* G2019S enhances clearance of *P. aeruginosa* lung infection. **(A)** Survival of young B6, WTOE, or G2019S mice after *P. aeruginosa* infection administered intranasally. **(B)** Real time monitoring of colonization and localization of bioluminescent bacteria in young B6, WTOE, or G2019S infected animals. Mice were imaged at 24 h after infection. The color bars indicate the intensity of the bioluminescence output, with red and blue denoting the high and low signals, respectively. **(C)** Survival of old B6, WTOE, or G2019S mice after *P. aeruginosa* infection. **(D)** Bacterial load in the lung homogenate of old infected mice 48 h after infection measured by CFU per gram of lung tissue. Each symbol represents the readout from a single mouse. Survival results are represented by Kaplan-Meier survival curves and were analyzed by log rank test. Sample sizes are indicated in the figure. Male and female mice are represented equally. One-way ANOVA with Tukey’s *post hoc* for multiple comparisons was used to compare across genotypes. Letters above groups indicate *post hoc* results. Groups that share the same letter were not significantly different from each other.

To assess whether *Lrrk2* G2019S modifies responses to acute mucosal gram-negative bacterial challenge, we inoculated male and female mice with bioluminescent *P. aeruginosa* PAO1 P1-lux intranasally. This model produces an acute pneumonia with significant monocyte infiltration into the lung. Age-matched 3–4 month old B6, WTOE and G2019S mice were monitored for survival and bacterial clearance. While significance was not achieved due to low sample size, G2019S mice were the only survivors compared to B6 and WTOE controls 72 h after exposure. Remarkably, G2019S mice had a faster loss of bacterial signal after infection (Figures 1A, B). Bioluminescent tracking indicated that this protection may have been due to increased clearance of the bacteria after infection (Figure 1B).

Because the penetrance of the *Lrrk2* BAC overexpression increases as a function of age (Yao et al., 2022), we repeated the experiment in aged (20–22 month-old) animals and found that this phenotype persisted in aged mice. G2019S mice survived better than B6 controls with WTOE trending in the same direction. Both overexpressing genotypes showed reduced bacterial burden in the lung lavage (Figures 1C, D). These data indicate that *Lrrk2* overexpression can promote host defense. In both young and old age, G2019S confers an immunological advantage by triggering a more efficient clearance of acute gram-negative *P. aeruginosa* infection relative to wildtype *Lrrk2*.

### 3.2 G2019S aggravates mortality in polymicrobial sepsis through hematopoietic cells

**FIGURE 2:**
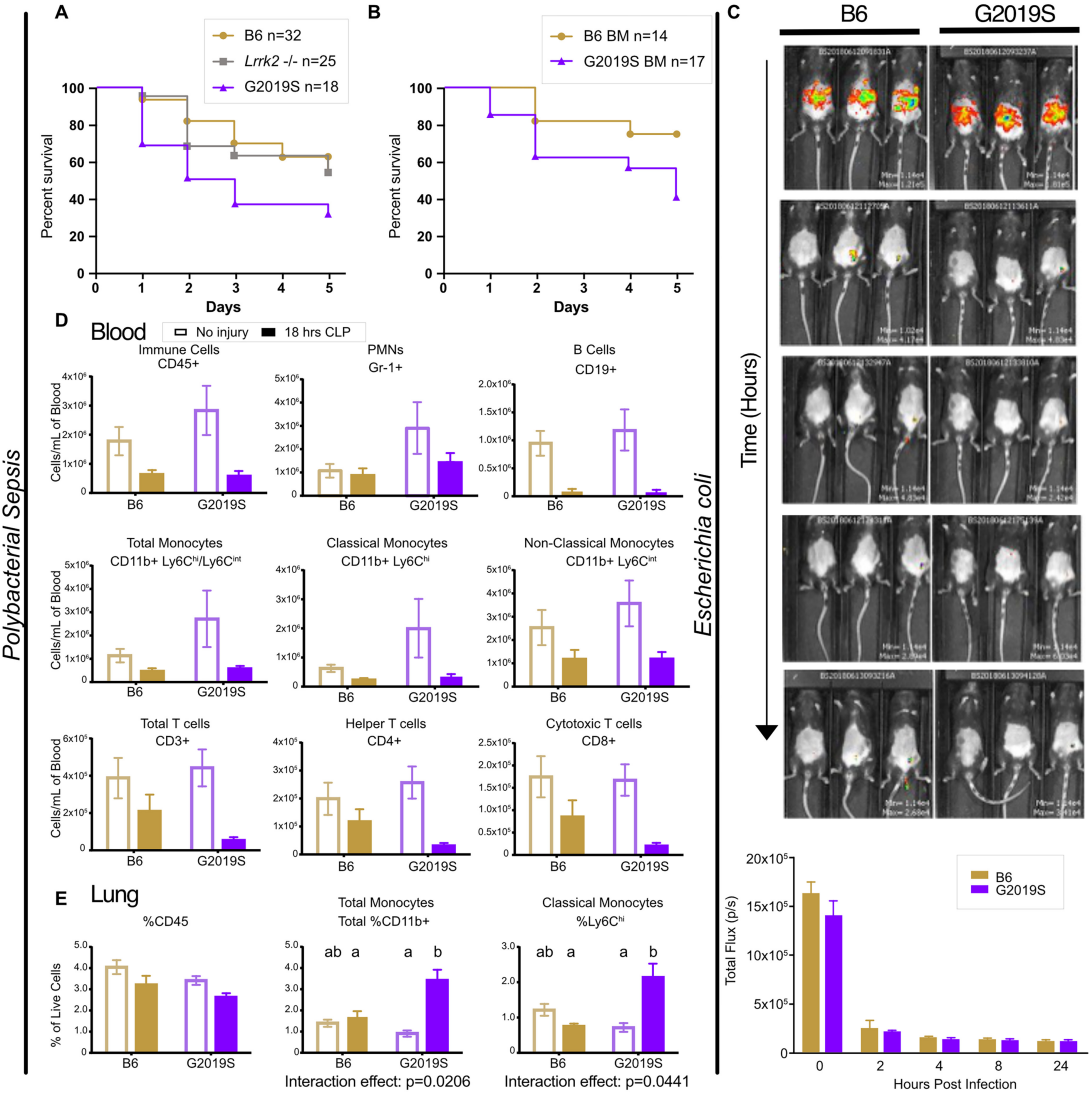
*Lrrk2* G2019S worsens polymicrobial sepsis through hematopoietic cells. **(A)** Survival curve of B6, G2019S, and Lrrk2^–/–^ animals after cecal ligation puncture to induce sepsis. **(B)** Bone marrow (BM) from either G2019S or B6 mice was transferred to B6 recipient mice. Survival curves of the recipient mice after CLP are displayed. **(C)** Images and quantification of bioluminescent *E. coli* delivered i.p. to B6 or G2019S mice. **(D)** Flow cytometric quantification of circulating CD45^+^ immune cells, peripheral mononuclear cells (PMNs), CD19^+^ B cells, total monocytes, classical monocytes, and non-classical monocytes, total T cells, CD4^+^ T helper cells, and CD8^+^ effector T cells in the blood of B6 and G2019S mice 18 h after CLP. **(E)** Quantification of the proportion of total immune cells, total monocytes, and total classical monocytes in the lung homogenate of B6 and G2019S mice 18 h after CLP. Sample sizes are indicated in the figure. Male and female mice are represented equally. Two-way ANOVA with Tukey’s *post hoc* for multiple comparisons was used to compare across genotypes. Letters above groups indicate *post hoc* results. Groups that share the same letter were not significantly different from each other.

We next asked whether the same genotype confers protection in a systemic infection setting using the cecal ligation and puncture (CLP) model of polymicrobial sepsis. This model elicits a robust and early innate response driven by neutrophil and macrophage activation to mostly gram-negative bacteria. In contrast to the *P. aeruginosa* model, G2019S mice showed increased mortality compared with *Lrrk2*^–/–^ and B6 animals (Figure 2A).

To determine whether this phenotype reflected a hematopoietic cell-intrinsic effect, we reconstituted irradiated CD45.1 recipients with bone marrow from G2019S or control donors (Supplementary Figure 2). Recipients of G2019S bone marrow also showed increased mortality after CLP, indicating that the survival defect is driven by bone marrow-derived cells from the overexpressing G2019S *Lrrk2* mice (Figure 2B).

To test whether this reflected a generalized defect in bacterial clearance, we infected mice with bioluminescent Xen14 *E. coli* through an intraperitoneal injection. No genotype-dependent differences in bacterial signal were detected over 24 h (Figure 2C), suggesting that the septic phenotype is not explained by altered clearance of this organism alone.

In order to better understand the differential response to CLP between genotypes, we conducted flow cytometric analysis on the blood. Flow cytometric analysis 18 h after CLP showed no genotype-dependent differences in circulating leukocyte populations (Figure 2D) or plasma cytokines (Supplementary Figure 3), but lungs from G2019S mice contained a higher proportion of CD11b^+^ cells and Ly6C^hi^ inflammatory monocytes (Figure 2E). This is a hallmark of acute respiratory distress syndrome commonly seen in late-stage sepsis (Dang et al., 2022). Overall, these data indicate that there is a higher proportion of proinflammatory monocytes that infiltrate the lung tissue of G2019S mice during sepsis which may contribute to higher mortality of G2019S mice relative to B6. These findings are consistent with exaggerated myeloid recruitment to the lung during sepsis, a feature that may contribute to lung injury and poor survival.

### 3.3 G2019S alters non-classical monocyte populations during *L. monocytogenes* infection

**FIGURE 3:**
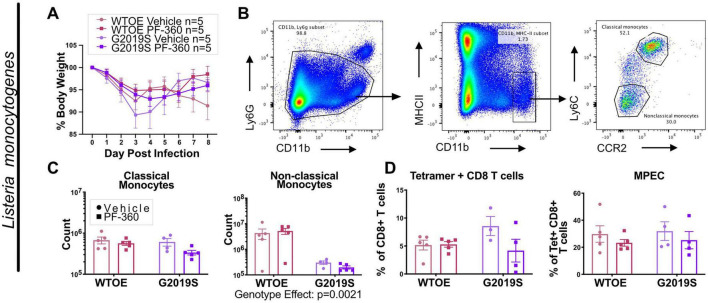
*Lrrk2* G2019S alters non-classical monocyte localization during *L. monocytogenes* infection. **(A)** Percent body weight of vehicle and PF-360 treated *L. monocytogenes* inoculated mice compared across genotypes until 8-days post-infection. **(B)** Gating strategy for classical and non-classical monocytes from the spleen. **(C)** Number of classical and non-classical monocytes in the spleen of *L. monocytogenes-*infected mice 8-days post infection. **(D)** Number of *L. monocytogenes* antigen-specific CD8^+^ cells and MPECs (KLRG-1^–^, CD127^+^) isolated from the spleen 8 days after infection. Sample sizes are indicated in the figure. Male and female mice are represented equally. Two-way ANOVA with Tukey’s *post hoc* for multiple comparisons was used to compare across genotypes. Letters above groups indicate *post hoc* results if ANOVA revealed genotype effects. Groups that share the same letter were not significantly different from each other.

To probe responses to intracellular bacterial infection and antigen presentation, we challenged WTOE and G2019S mice with intravenously administered *L. monocytogenes*. We treated groups with the selective LRRK2 kinase inhibitor PF-360 to determine whether inhibition of LRRK2 kinase activity modified the observed phenotypes, thereby assessing the contribution of kinase-dependent signaling to disease manifestations. We confirmed that the LRRK2 kinase inhibitor PF-360 efficiently reduced the phosphorylation of LRRK2 both *in vitro* and *in vivo* through oral gavage (Supplementary Figure 4). *L. monocytogenes* infection led to weight loss, which was comparable across groups, indicating similar overall disease severity (Figure 3A).

Eight days after infection, splenic monocyte subsets were quantified by flow cytometry. Classical monocyte numbers were unchanged, whereas non-classical monocytes were increased in WTOE mice relative to G2019S mice, including under kinase inhibition (Figures 3B, C). These data suggest that G2019S selectively reduces non-classical monocytes rather than broadly impairing monocyte abundance. Consistent with our previous findings (Figures 2E, F), these data suggest that non-classical monocytes may be trafficked out of the spleen and into tissues more efficiently in G2019S than in WTOE mice in a kinase-independent manner. Migration, retention, and chemokine-response assays will be necessary to confirm these observations.

Examination of antigen-specific CD8^+^ effector T cells and memory precursor effector cells (MPECs) revealed no differences between groups (Figure 3D). Together, these data indicate that LRRK2 G2019S reshapes monocyte dynamics during intracellular bacterial infection without measurably altering clinical disease progression in this model.

### 3.4 G2019S modulates CD8^+^ T cell distribution during influenza and LCMV infection

**FIGURE 4:**
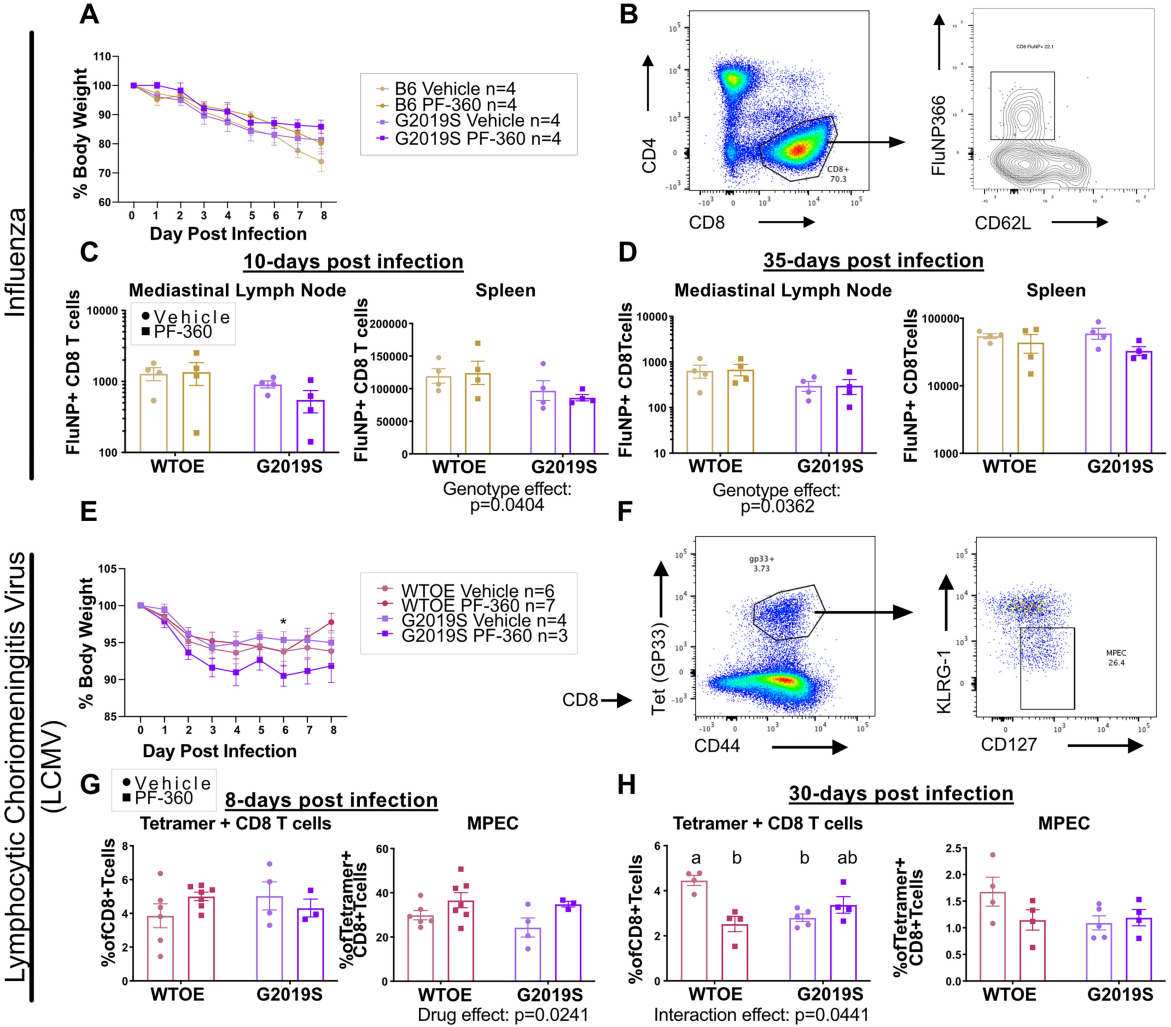
*Lrrk2* G2019S modulates CD8^+^ T cell responses during influenza and LCMV infection. **(A)** Percent body weight of vehicle and PF-360-treated influenza inoculated mice compared across genotypes until 8-days post infection. **(B)** Gating strategy used to identify influenza antigen-specific T cells (FluNP366^+^CD8^+^ T cells). Number of influenza antigen-specific T cells in the mediastinal lymph node and spleen at **(C)** ten or **(D)** thirty-five days post-infection. **(E)** Percent body weight of PF-360 and vehicle treated LCMV inoculated mice compared across genotypes until 8-days post-infection (effect of time effect of time: F(8, 235) = 14.60; *p* < 0.0001) and G2019S (interaction effect: F(8, 202) = 2.173; *p* = 0.0309) Asterisk (*) significates a statistically significant difference between vehicle and PF-360 treated G2019S mice on day 6. **(F)** Gating strategy used to identify LCMV antigen-specific T cells (TetGP33^+^ CD8^+^) and MPECs (KLRG-1^–^, CD127^+^). **(G**) Early effector and memory precursor responses in spleen at day 8. **(H)** Memory-phase responses in spleen at day 30. Sample sizes are indicated in the figure. Male and female mice are represented equally. Two-way ANOVA with Tukey’s *post hoc* for multiple comparisons was used to compare across genotypes for bar graphs. Groups that share the same letter were not significantly different from each other.

To extend our findings that G2019S overexpression modulates innate immune responses including bacterial clearance and monocyte localization (Figures 1–3), we next aimed to determine how G2019S overexpression and its associated increased kinase activity would influence the type I adaptive immune response, T cell activity, and cross presentation. First, we examined whether *Lrrk2* G2019S influences antiviral adaptive immunity using influenza virus to model pulmonary CD4 and antibody-mediated immunity. This model efficiently tests T cell priming and homing to the draining lymph nodes of the lung, the mediastinal lymph nodes. B6 and G2019S mice were inoculated with H1N1 intranasally and treated with PF-360 or vehicle twice daily for 14 days after inoculation. To identify whether there was a modulation of the T cell response irrespective of the clinical presentation, we used flow cytometry to identify FluNP-specific CD8^+^ T cells. Following intranasal influenza A infection, weight loss was similar across genotypes and PF-360 treatment groups (Figure 4A).

At day 10 after infection, FluNP-specific CD8^+^ T cells were not changed in the mediastinal lymph node, but G2019S mice showed fewer antigen-specific CD8^+^ T cells in the spleen regardless of kinase inhibition (Figures 4B, C). By day 35, the pattern shifted, with fewer FluNP-specific CD8^+^ T cells in the mediastinal lymph node and no spleen differences (Figure 4D). Overall, these data are most consistent with altered LRRK2 kinase-independent T cell disruption rather than a profound defect in priming. Additional studies examining target PF-360 engagement in T cells will be required to confirm LRRK2 kinase-independent T cell disruption.

To further assess T cell priming and memory we next tested LCMV, a virus which usually causes mild symptoms in healthy humans but leads to severe respiratory illness and meningitis in mice (Bocharov et al., 2015). LCMV infection provides a stringent measure of cytotoxic T cell priming and memory formation. In contrast to the influenza model, LCMV leads to systemic, chronic infection, rather than acute and cleared infection. No differences in weight loss were observed between groups (Figure 4E). In the early phase of infection, genotype-dependent differences in tetramer-positive CD8^+^ T cells were minimal, although PF-360 increased memory precursor effector cells at day 8 (Figures 4F, G). At day 30, vehicle-treated WTOE mice contained more LCMV-specific CD8^+^ T cells in the spleen than either G2019S mice or WTOE mice treated with PF-360, whereas memory precursor effector cells were not significantly different among groups (Figure 4H). Clonal expansion peaks at eight days after LCMV infection and so our data indicate that there was no *Lrrk2*-mediated modulation of CD8^+^ T cell clonal expansion. Further, there was no kinase- or genotype-dependent change in T cell maturation into MPECs. Based on these data, we conclude that *Lrrk2* modulation does not affect antigen presentation, cross-presentation, CD8 clonal expansion, or transition to MPECs. However, the G2019S mutation may modulate CD8 T cell localization. Taken together, these findings indicate that *Lrrk2* G2019S does not grossly impair antiviral clonal expansion, but it does alter the localization and maintenance of antigen-specific CD8^+^ T cells in a manner that is not fully reversed by kinase inhibition.

## 4 Discussion

Our findings demonstrate that *Lrrk2* G2019S does not impose a uniform immune phenotype but instead alters host defense responses in a pathogen- and tissue-dependent manner. Across multiple infection models, *Lrrk2* G2019S overexpression was associated with divergent outcomes: improved bacterial clearance and survival during localized *P. aeruginosa* lung infection, but increased mortality in polymicrobial sepsis. These contrasting phenotypes suggest that LRRK2 activity does not simply amplify or suppress inflammation globally, but rather modulates the coordination and localization of immune responses, which can be beneficial or detrimental depending on the infectious context, consistent with previous work from our group (Hughes et al., 2025).

Our novel findings have two important implications. First, they provide a mechanistic explanation for why the same variant can be protective in one infectious setting but harmful in another. In the *P. aeruginosa* pneumonia model, enhanced bacterial clearance and improved survival in G2019S and WTOE mice indicates that increased LRRK2 activity can support effective host defense in a localized mucosal setting. In contrast, in the CLP model of polymicrobial sepsis, G2019S *Lrrk2* overexpression expression was associated with increased mortality and enhanced accumulation of CD11b^+^ and Ly6C^hi^ myeloid cells in the lung. While these findings are consistent with exaggerated inflammatory recruitment contributing to tissue pathology, our data do not directly establish causality. Instead, they suggest that the spatial distribution and regulation of inflammatory cells, rather than their absolute magnitude alone, may be a key determinant of infection outcome. Second, they suggest that LRRK2-directed therapeutics will need to balance potential benefits in infection control with effects on host defense. Over time, repeated or poorly resolved inflammatory episodes of this nature could contribute to systemic inflammatory burden.

The current study does not identify the precise cell types and cellular pathways downstream of LRRK2 that shape innate and adaptive immune cell behavior. Future work using conditional or knock-in models to direct cell type specificity coupled with mechanistic assays will be necessary to define how LRRK2 kinase activity intersects with cytoskeletal dynamics, trafficking and inflammatory signaling. Specifically, the bone marrow chimera experiments further support a hematopoietic cell–intrinsic contribution to the septic phenotype. However, the specific cell types and molecular pathways responsible remain to be defined. LRRK2 has been implicated in cytoskeletal dynamics, vesicular trafficking, and innate immune signaling pathways, all of which influence leukocyte migration, retention, or effector function (Usmani et al., 2021). Although our data are consistent with altered immune cell trafficking or positioning, we did not directly measure migratory behavior or chemokine responsiveness, and therefore these interpretations should be considered hypothesis-generating and will need to be investigated empirically. The *L. monocytogenes* model provided additional support for selective modulation of myeloid subsets, as G2019S mice exhibited reduced non-classical monocytes in the spleen without changes in disease severity. This finding is consistent with altered monocyte distribution rather than a global defect in monocyte production or survival. However, without direct tracking or functional assays, we cannot distinguish between altered trafficking, retention, or differentiation. Future studies using fate mapping or *in vivo* migration assays will be necessary to resolve these possibilities. In viral infection models, G2019S expression did not significantly alter weight loss or overt disease severity, but rather it was associated with changes in the distribution of antigen-specific CD8^+^ T cells between lymphoid compartments. Importantly, we did not observe consistent defects in clonal expansion or differentiation into memory precursor effector cells. These findings suggest that *Lrrk2* G2019S may influence T cell localization or maintenance rather than priming or expansion. As with the myeloid phenotypes, these conclusions are based on endpoint measurements, and direct assessment of trafficking or tissue residency will be required to confirm this interpretation.

A key question is whether the observed phenotypes are dependent on increased LRRK2 kinase activity. Pharmacologic inhibition with PF-360 effectively reduced LRRK2 phosphorylation, but did not uniformly reverse all phenotypes, suggesting that some effects of G2019S may be partially independent of kinase activity or incompletely suppressed by pharmacologic inhibition. However, given the limitations of inhibitor-based approaches—including potential variability in tissue penetration and incomplete pathway suppression—our data do not definitively resolve the relative contributions of kinase-dependent versus kinase-independent mechanisms. Genetic approaches will be required to address this question more rigorously.

Several limitations of this study warrant consideration. First, both sexes were used in the analysis of these models to better understand gross physiological risk factors associated with LRRK2 overactivity. However, sex-specificity of immune activity has been well documented and further examination of these antibacterial responses should be studied in the context of sex (Di Florio et al., 2020). Similarly, we observed age-dependent changes in susceptibility in *P. aeruginosa* infection but did not test age as a variable in other infection models. Likely, age-related changes LRRK2 activity may compound the effects of G2019S mutation. Second, the overexpression system used here may not fully recapitulate endogenous regulation in human mutation carriers, and some phenotypes may be influenced by age, sex or tissue compartment. Despite these limitations, our findings support a model in which *LRRK2* G2019S reshapes immune responses by altering how immune cells are deployed across tissues, rather than simply increasing or decreasing their activity. This framework may help explain why the same genetic variant can be protective in some infectious contexts while detrimental in others. More broadly, these data highlight the importance of considering context-dependent immune regulation when evaluating the role of LRRK2 in disease, including the PD-associated *LRRK2* G2019S variant.

In summary, our findings support a model in which LRRK2 shapes the spatial and contextual deployment of immune responses rather than their magnitude alone, offering a new perspective on how genetic variation can produce opposing outcomes across disease settings. In the context of PD, it advances our understanding of how genetic risk for PD may intersect with lifetime inflammatory exposures and position dysregulated innate immunity as a potential contributor to neuronal vulnerability and a novel therapeutic target for intervention. Given the growing interest in LRRK2 kinase inhibitors as therapeutic agents for LRRK2-PD, our findings also underscore the need to better understand how modulating LRRK2 activity may influence host defense. While our data do not directly link infection responses to neurodegeneration, they suggest that genetically encoded differences in immune regulation could shape an individual’s response to environmental challenges over time. Defining the mechanisms by which LRRK2 regulates immune cell behavior across tissues will be essential for understanding both its role in infection biology and its potential contribution to disease risk.

These data identify LRRK2 as a regulator of context-dependent immune responses, revealing that a common PD–associated mutation can differentially shape host defense across infection settings. These findings highlight the importance of immune context in determining the consequences of genetic risk factors and have implications for the therapeutic targeting of LRRK2. Specifically, by demonstrating that LRRK2 G2019S produces divergent outcomes across infectious contexts, this study underscores the need to consider host defense when targeting LRRK2 therapeutically and provide a novel framework for understanding how genetic variation in immune regulation may influence disease risk and treatment responses.

## Data Availability

The raw data supporting the conclusions of this article will be made available by the authors, without undue reservation.
