# Supplementary material for: LRRK2 G2019S reprograms innate and adaptive immunity to drive context-dependent host defense outcomes
Source: Front Cell Neurosci. 2026 Jul 29;20:1860967. doi: 10.3389/fncel.2026.1860967 (PMC13461538; doi:10.3389/fncel.2026.1860967)
Supplement: Supplementary file 1 [file Supplementary_file_1.docx]

Supplementary Material

# 1 Supplementary Figures

# Supplementary Figure 1. Flow cytometry gating strategies for Figure 2.

# Supplementary Figure 2. Blood chimerism of bone marrow transplant recipients. Reconstitution of blood leukocytes post BMT was evaluated by flow cytometry on blood obtained from retro orbital puncture. Blood leukocyte repopulation was >90 % in all transplanted mice. After BMT, 94.3±0.7 % and 93.5±0.8 % of the blood leukocytes in mice receiving nontransgenic (nTg) and G2019S bone marrow respectively were CD45.2/ Ly5.2 (donor) positive; % reconstitution expressed as % of total live CD45^+^ cells

**Supplementary Figure 3. Plasma cytokines reveal genotype independent inflammation after polymicrobial sepsis.** Comparison of plasma cytokine levels from G2019S and B6 mice 18 hours post CLP or sham/No CLP groups are shown. Cytokine levels are represented as picograms/mL (pg/mL). Data values indicate group mean ±S.E.M; n= 5-9 mice/group. Statistically significant differences (p≤0.05; 1 way-*ANOVA* with Tukey’s multiple comparison test) between groups are shown as *. IL: interleukin; IFN-ɣ: interferon- gamma; TNF: tumor necrosis factor; KC-GRO: Keratinocyte chemoattractant - growth-regulated oncogene or chemokine C-X-C motif ligand 1.

**Supplementary Figure 4. PF-360 inhibits LRRK2 kinase activity in G2019S mice.** (A) Kidney and (B) lung protein lysates of G2019S mice after 7.5 mg/kg PF-360 oral gavage. Western blot showing phosphorylation of LRRK2 at p1292 at 6 hours after gavage and a time course of p5935 LRRK2 and pRab10 at several time points after gavage of PF-360.
